# Down-regulation of EZH2 genes targeting RUNX3 affects proliferation, invasion, and metastasis of human colon cancer cells by Wnt/β-catenin signaling pathway

**DOI:** 10.18632/aging.205197

**Published:** 2023-12-02

**Authors:** Guanli Liang, Lei Han, Ming Qu, Jun Xue, Dandan Xu, Xueliang Wu, Yonggang Lu

**Affiliations:** 1 Department of General Surgery, The First Affiliated Hospital of Hebei North University, Zhangjiakou 075000, China; 2 Institute of Oncology, The First Affiliated Hospital of Hebei North University, Zhangjiakou 075000, China; 3 Central Laboratory, The First Affiliated Hospital of Hebei North University, Zhangjiakou 075000, China; 4 Clinical Laboratory, Hebei General Hospital, Shijiazhuang, Shijiazhuang 05000, China

**Keywords:** EZH2, RUNX3, HCT116, HT29, Wnt/β-catenin signaling pathway

## Abstract

In order to detect the effect of EZH2 genes on proliferation, migration, invasion, and apoptosis of colon carcinoma cell strains HCT116 and HT29 by the Wnt/β-catenin signaling pathway, qRT-PCR was applied to measure relative expressions of EZH2, RUNX3, CEA, CA199, MMP-9, VEGF, β-catenin, and CyclinD1 in each group; Western-blot was employed with the intention of exploring relative expressions of these proteins *in vivo* and *in vitro*; monoclonal proliferation experiments and CCK-8 assay was adopted so as to check cell proliferation; the effect on cell migration was investigated via Transwell assay and cell scratch wound assay; flow cytometry was applied with a view to determining the effect on cell apoptosis. Transfected HCT116 cells are injected subcutaneously into nude mice. In colon cell strains HCT-116 and HT29, contrasted to the si-NC group, the RUNX3 expression was prominently up-regulated in the si-EZH2 group. Besides, expressions of CEA, CA199, MMP-9, and VEGF were significantly reduced; down-regulation of EZH2 genes remarkably inhibited cell proliferation, invasion and migration when facilitating apoptosis; down-regulation of EZH2 genes also significantly reduced expressions of essential proteins β-catenin and CyclinD1 on the Wnt pathway. The subcutaneous tumor body of nude mice was reduced. EZH2-OE is the opposite trend to si-EZH2; The EZH2 gene may target regulatory RUNX3 regulation via that Wnt/β-catenin signaling pathway, hence affecting colon carcinoma cell proliferation, invasion, migration, and apoptosis. Therefore, EZH2 may become a promising target for the clinical therapy of colon carcinoma.

## INTRODUCTION

In the light of the new data, there were 19.3 million cancer cases diagnosed globally in 2020, of which about 2 million were colorectal cancer cases, making it the third most common cancer after breast cancer and lung cancer and having the second highest mortality rate [1]. Due to a lack of specific early diagnosis techniques, when diagnosed most of the patients who suffer from colorectal carcinoma are entering into the mid-term or advanced stages when diagnosed, making surgery an unfeasible option for them [2]. Therefore, it is essential to explore the pathogenesis of colorectal cancer and find effective and precise diagnostic and therapeutic methods.

The drosophila gene enhancer of zeste homolog 2 (EZH2) affects the expressions of target gene histones by promoting their methylation [3]. In the hypoxic tumor micro-environment, the activity of target gene histones rises, which is associated with the silencing of tumor suppressor genes as well as the tumor emergency and progress [4]. Human runt-associated transcription factor 3 (RUNX3) becomes an anti-oncogene involved in regulating cell proliferation, differentiation, alongside apoptosis. Previous researches have proved that EZH2 is capable of regulating cell proliferation in larynx carcinoma through targeting RUNX3 via the Wnt /β-catenin signaling pathway [5]; EZH2 inhibitors are capable of activating RUNX3 and up-regulating the expressions of SETDB1 and ΔNp63α, thus antagonizing the phenotype of esophageal squamous cell carcinoma [6]. Apart from EZH2 and RUNX3 genes expressions, proteins from colon cancer tissues and healthy tissues were histologically examined by our project group using immunohistochemical method and real time fluorescence quantitative reverse transcription-polymerase chain effect. Our findings revealed significant differences in both EZH2 and RUNX expression, while also confirming a significant negative correlation between their expressions in colon cancer tissues [7]. However, the mechanism of action through which EZH2 targets and regulates RUNX3 expression and the resulting variations in various behaviors presented in cancer cell life activities have not been elucidated. Tumor proliferation and differentiation are connected to abnormally activating that pathway. Additionally, β-catenin is an essential element in the above-mentioned pathway, while CyclinD1 is an essential target gene of the downstream pathway. In our research, EZH2 and RUNX3 expressions in colon carcinoma cell strains were examined at the cytological level, verified the effects of EZH2 upon their migration, proliferation, apoptosis, and invasion were verified, and the molecular mechanism of EZH2 action was further explored so as to provide new targets for targeted therapy of colon cancer.

## RESULTS

Stable si-EZH2 transfected cells were created, and the knockdown efficiency of si-EZH2-3 (85.54 ± 3.67)% was significantly higher than that of si-EZH2-1 (69.75 ± 4.82)% and si-EZH2-2 (62.11 ± 1.62)%. Therefore, si-EZH2-3 was used for subsequent experiments (Supplementary Figure 1).

### qRT-PCR results

**Figure 1:**
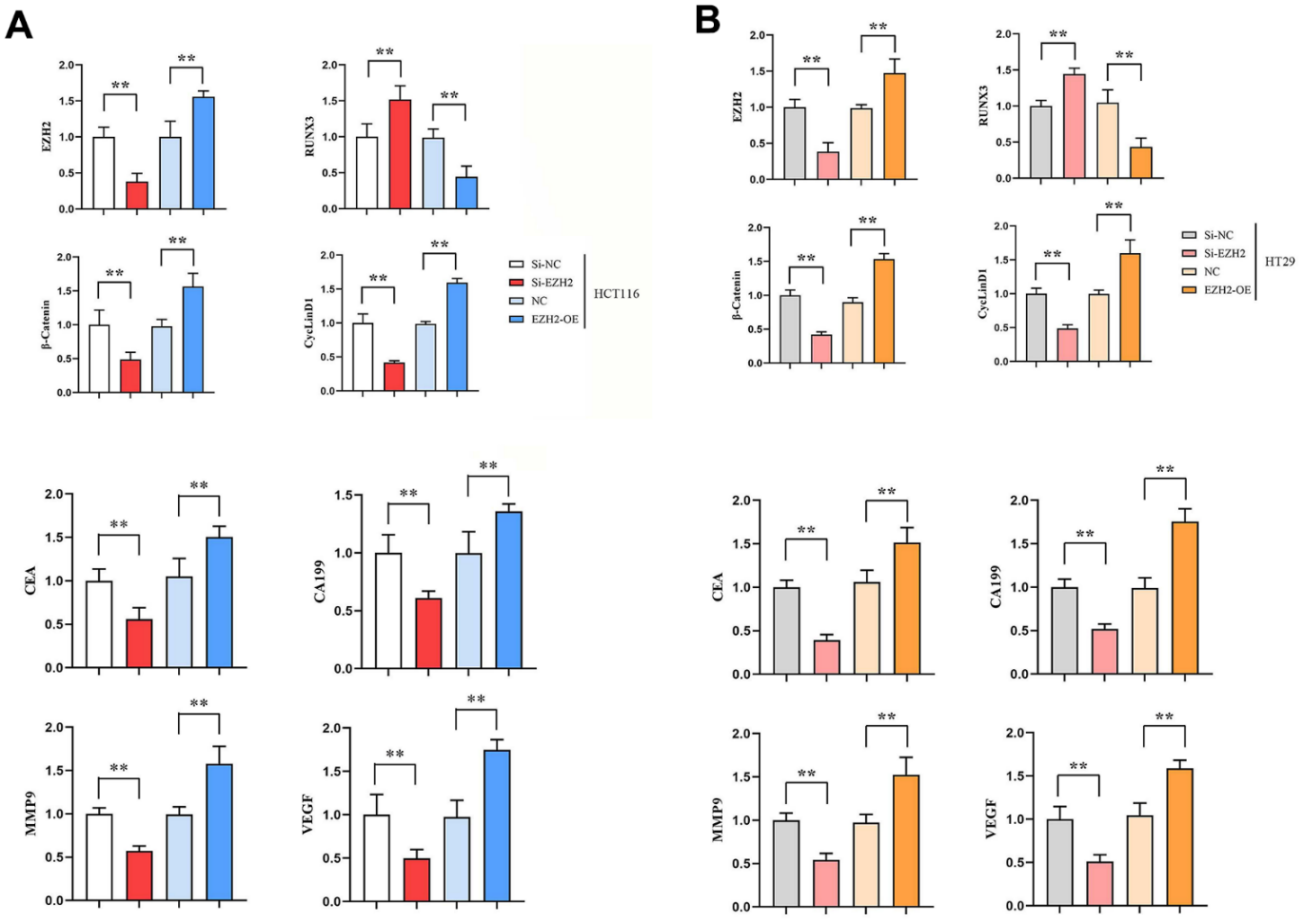
**Effect of transfection in colon cancer cells HCT116, HT29 on the expressions of EZH2, RUNX3, CEA, CA199, MMP-9, VEGF, β-catenin and CyclinD1 genes.** (**A**) Relative expression of EZH2, RUNX3, CEA, CA199, MMP-9, VEGF, β-catenin and CyclinD1 genes in HCT116 cells; (**B**) Relative expression of EZH2, RUNX3, CEA, CA199, MMP-9, VEGF, β-catenin and CyclinD1 genes in HT29 cells. (**P < 0.01; N=3/Group).

Expressions of EZH2 mRNA, β-catenin mRNA and CyclinD1 mRNA in si-EZH2 group were prominently lower than those in si-NC group. However, contrasted to NC group, expressions of EZH2 mRNA, β-catenin mRNA and CyclinD1 mRNA in EZH2-OE group were significantly higher. (*P* < 0.01); RUNX3 mRNA expression in si-EZH2 group prominently exceeded that in si-NC group, besides, RUNX3 mRNA expression in EZH2-OE group was prominently lower than that in the NC group (*P*<0.01); expressions of CA199 mRNA, CEA mRNA, MMP-9 mRNA and VEGF mRNA in si-EZH2 group were prominently lower than those in si-NC group, besides, in EZH2-OE group, their expressions prominently exceeded those in NC group (*P*<0.01); expressions of MMP-9 mRNA and VEGF mRNA in si-EZH2 group were prominently lower than those in the si-NC group, besides, expressions of MMP-9 mRNA and VEGF mRNA in EZH2-OE group prominently exceeded those in NC group (*P*<0.01) (Figure 1).

### Western blot results

**Figure 2:**
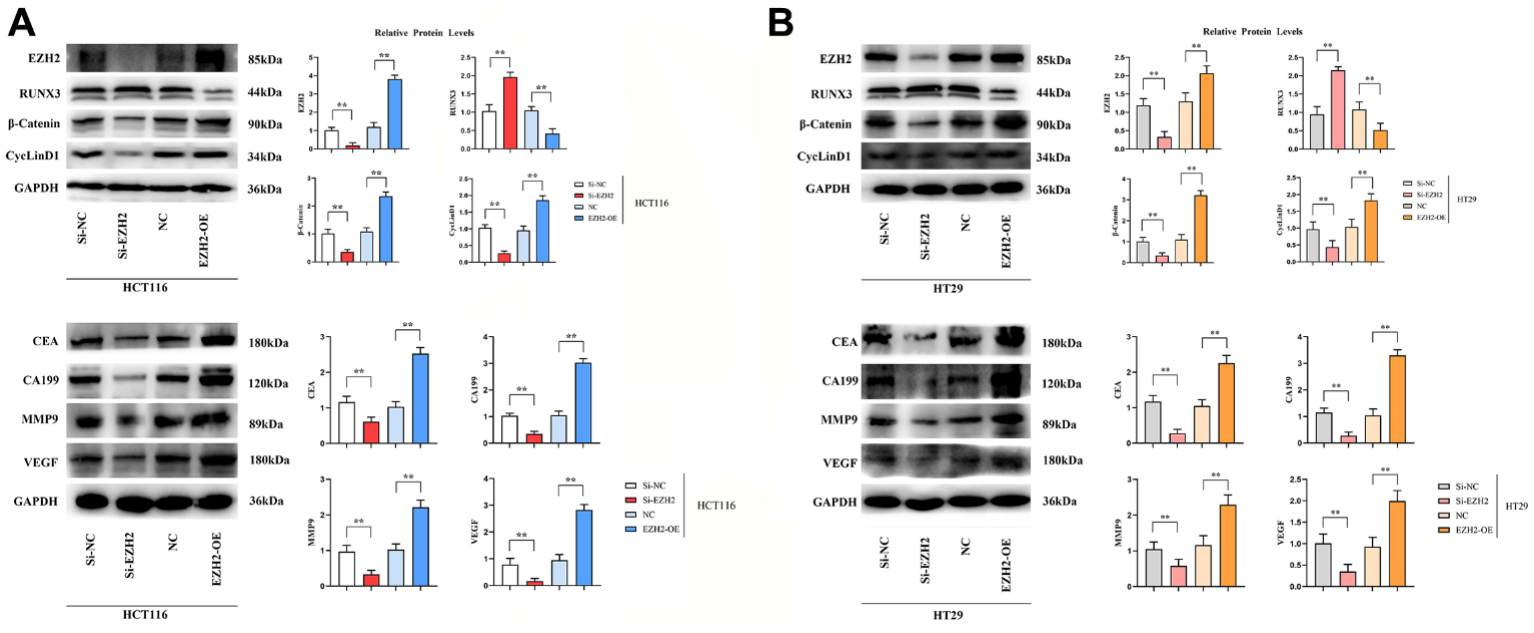
**Effect of si-EZH2 and EZH2-OE transfection in colon cancer cells HCT116 and HT29 on the expression of EZH2, RUNX3, CEA, CA199, MMP-9, VEGF, β-catenin and CyclinD1 proteins.** (**A**) Statistics on protein banding and relative protein expression of EZH2, RUNX3, CEA, CA199, MMP-9, VEGF, β-catenin and CyclinD1 in HCT116 cells; (**B**) Statistics on protein banding and relative protein expression of EZH2, RUNX3, CEA, CA199, MMP-9, VEGF, β-catenin and CyclinD1 in HT29 cells. (**P < 0.01; N=3/Group).

Expressions of EZH2 protein, β-catenin protein and CyclinD1 protein in si-EZH2 group were prominently lower than those in si-NC group, besides, expressions of EZH2 protein, β-catenin protein and CyclinD1 protein in EZH2-OE group prominently exceeded that in NC group. (*P* < 0.01); the RUNX3 expression in the si-EZH2 group prominently exceeded that in the si-NC group, whereas in EZH2-OE group, it was prominently lower in contrast to the NC group (*P* < 0.01); expressions of CEA protein, CA199 protein, MMP-9 protein and VEGF protein in the si-EZH2 group were prominently lower than those in the si-NC group, besides, expressions of CEA protein, CA199 protein, MMP-9 protein and VEGF protein in EZH2-OE group were prominently exceeded that in NC group (*P<*0.01); Contrasted to the si-NC group, expressions of MMP-9 protein and VEGF protein in the si-EZH2 group were prominently lower, and contrasted to NC group, expressions of MMP-9 protein and VEGF protein in EZH2-OE group were prominently more elevated (*P<*0.01) (Figure 2).

### Cell proliferation

The effect of EZH2 upon colon cancer cell proliferation was measured via CCK-8 assay. In HCT116 and HT29 cells, the OD values of si-EZH2 group at 12 h, 24 h and 36 h were prominently less than those of si-NC group, besides, the OD values of EZH2-OE group at 12 h, 24 h and 36 h prominently exceeded those of NC group (*P* < 0.01). It was shown in the CCK8 that EZH2 exerted a positive feedback effect upon colorectal cancer cell proliferation (Supplementary Figure 2).

### Cell migration

**Figure 3:**
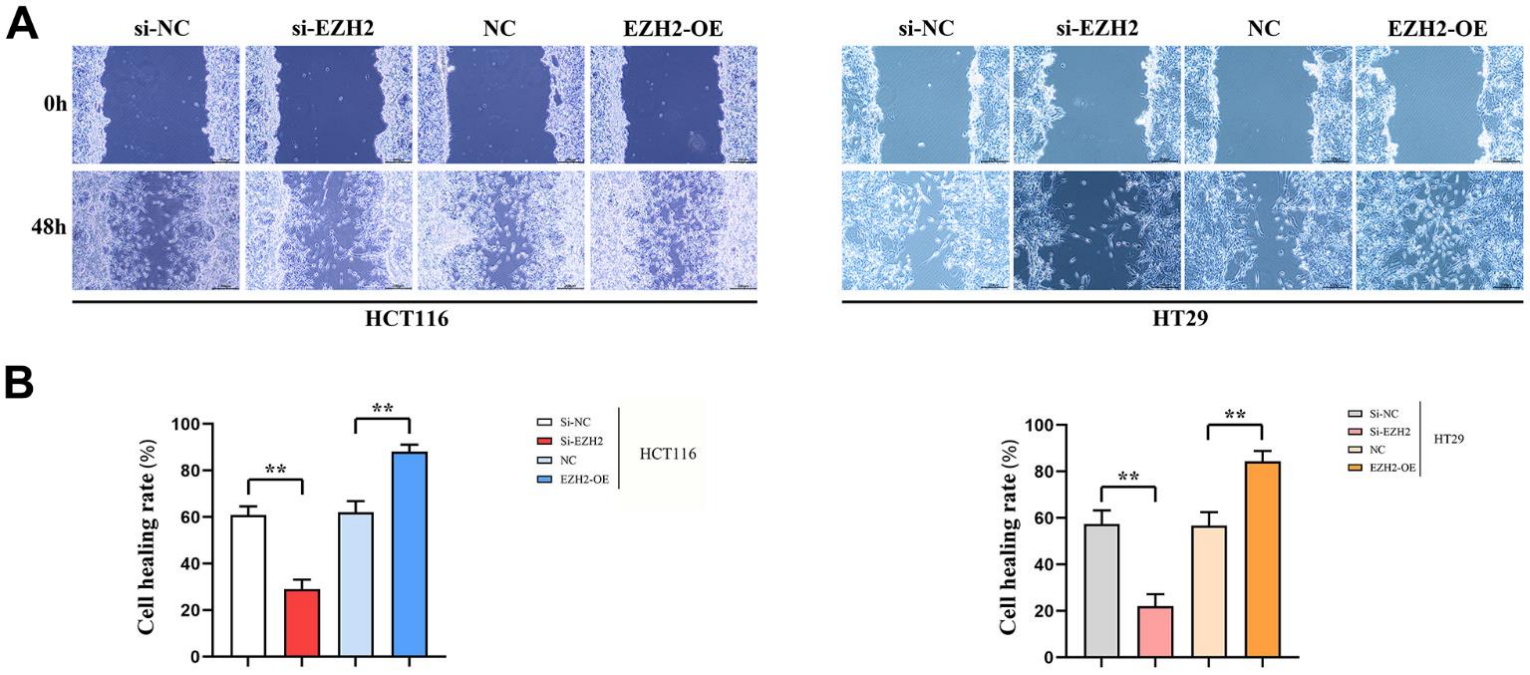
**Cell scratch healing rate.** (**A**) Cell scratch results plot for HT29 cells and HCT116 cells; (**B**) Data on cell healing rates of HT29 cells and HCT116 cells. (**P < 0.01; N=3/Group).

The healing rate in the si-EZH2 group after 48 h was prominently lower than that in the si-NC group; The healing rate in the EZH2-OE group after 48 h prominently exceeded that in NC group. A statistically distinction was seen between HCT116 and HT29 cells (*P* < 0.01). The cell scratch wound assay indicated that EZH2 has a positive feedback effect upon metastasis of colorectal carcinoma cells (Figure 3).

### Cell invasion

**Figure 4:**
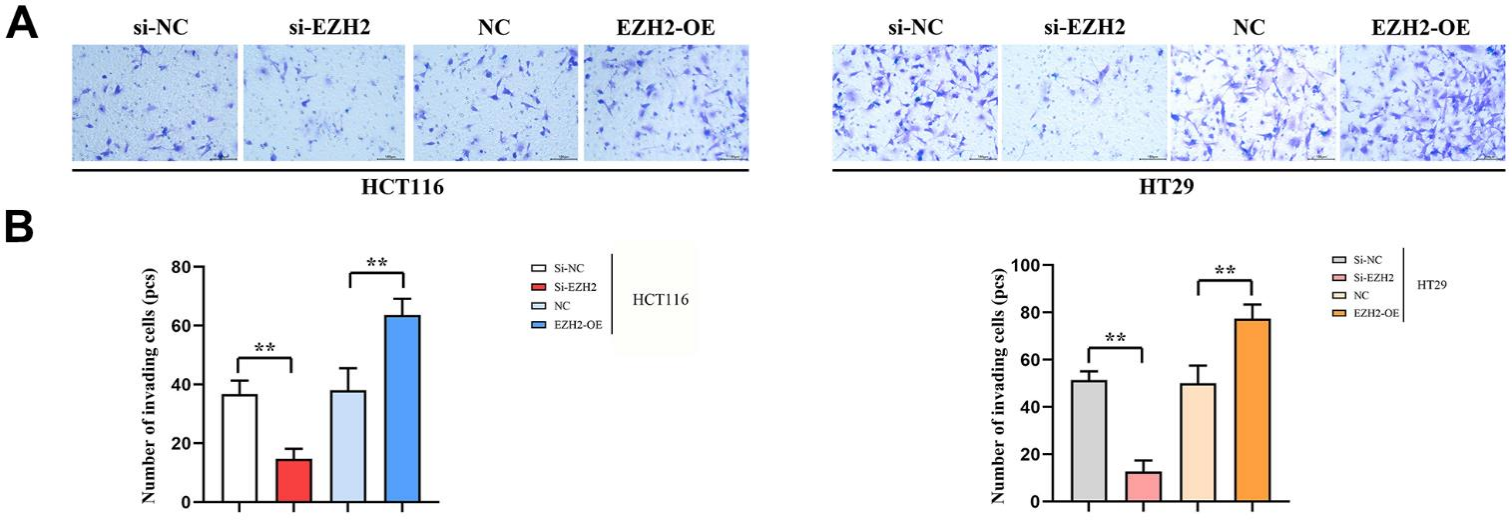
**Number of transmembrane cells (100PX).** (**A**) Invasion results plot of HT29 cells and HCT116 cells; (**B**) HT29 cell and HCT116 cell invasion statistics. (**P < 0.01 *P < 0.05; N=3/Group).

As for transferred cells, their number was figured out in Transwell migration and invasion assay, it was found that between HCT116 and HT29 cells, in the si-EZH2 group, the number was prominently less than that in si-NC group. Besides, in the EZH2-OE group, the number was exceeded that in the NC group. Furthermore, a statistical distinction was seen in both HCT116 and HT29 cells (*P<*0.05). It was shown in the consequences that EZH2 was in proportion to colorectal carcinoma cell invasion (Figure 4).

### Cell apoptosis

**Figure 5:**
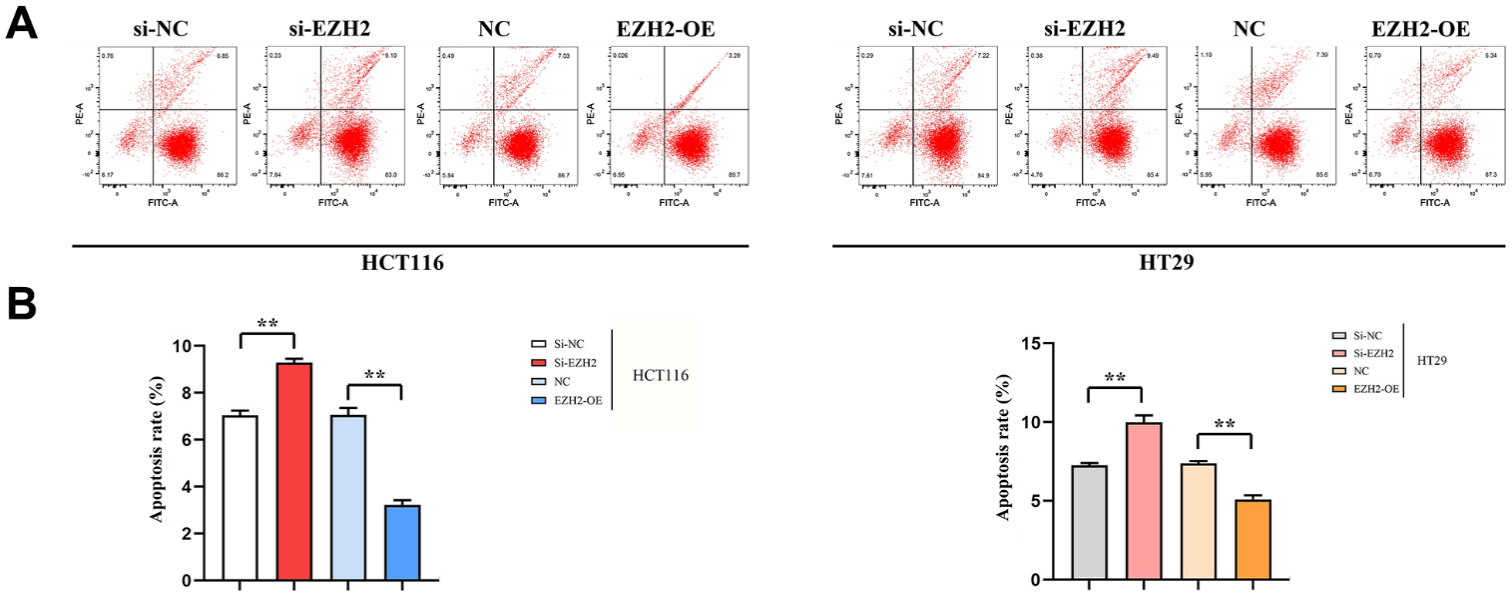
**Effect of EZH2 on early apoptosis of HCT116 and HT29 cells.** (**A**) Plot of apoptosis outcomes in HCT116 and HT29 cells; (**B**) Apoptosis data of HCT116 and HT29 cells. (**P < 0.01; N=3/Group).

The early apoptosis percentage in the si-EZH2 group prominently exceeded that in the si-NC group; however, early apoptosis rate in the EZH2-OE group prominently exceeded that in the NC group. A statistically distinction was seen in both HCT116 and HT29 cells (*P* < 0.01). The results illustrated that EZH2 was inversely proportional to the early apoptosis percentage of colorectal cancer cells (Figure 5).

### Results of monoclonal proliferation experiments

**Figure 6:**
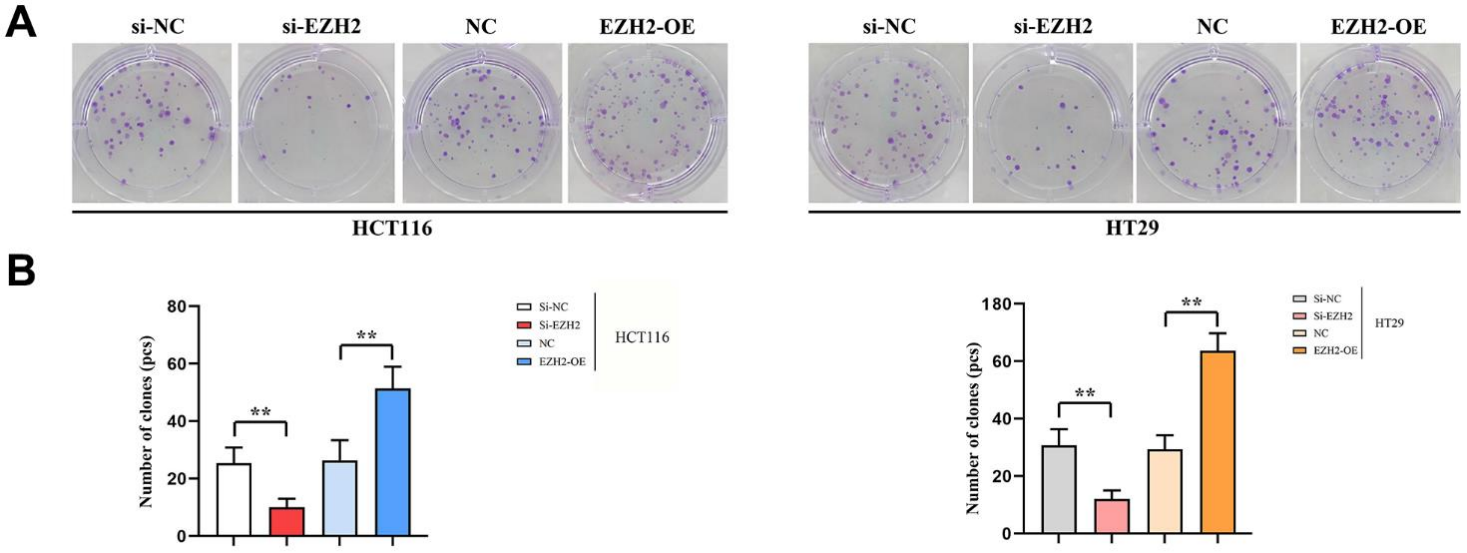
**Number of monoclonal cells.** (**A**) Plot of results of HCT116 cell and HT29 monoclonal experiments; (**B**) HCT116 cells and HT29 monoclonal experimental data statistics. (** *P* < 0.01, ns *P* > 0.05; N=3/Group).

In comparison to si-NC group, the quantity of clones in the si-EZH2 group was prominently reduced. While within EZH2-OE group, the number of clones remarkably exceeded that of the NC group. There existed prominent distinctions among values. It shows that EZH2 is capable of promoting colorectal carcinoma proliferation (Figure 6).

### Subcutaneous cancers among nude mice

**Figure 7:**
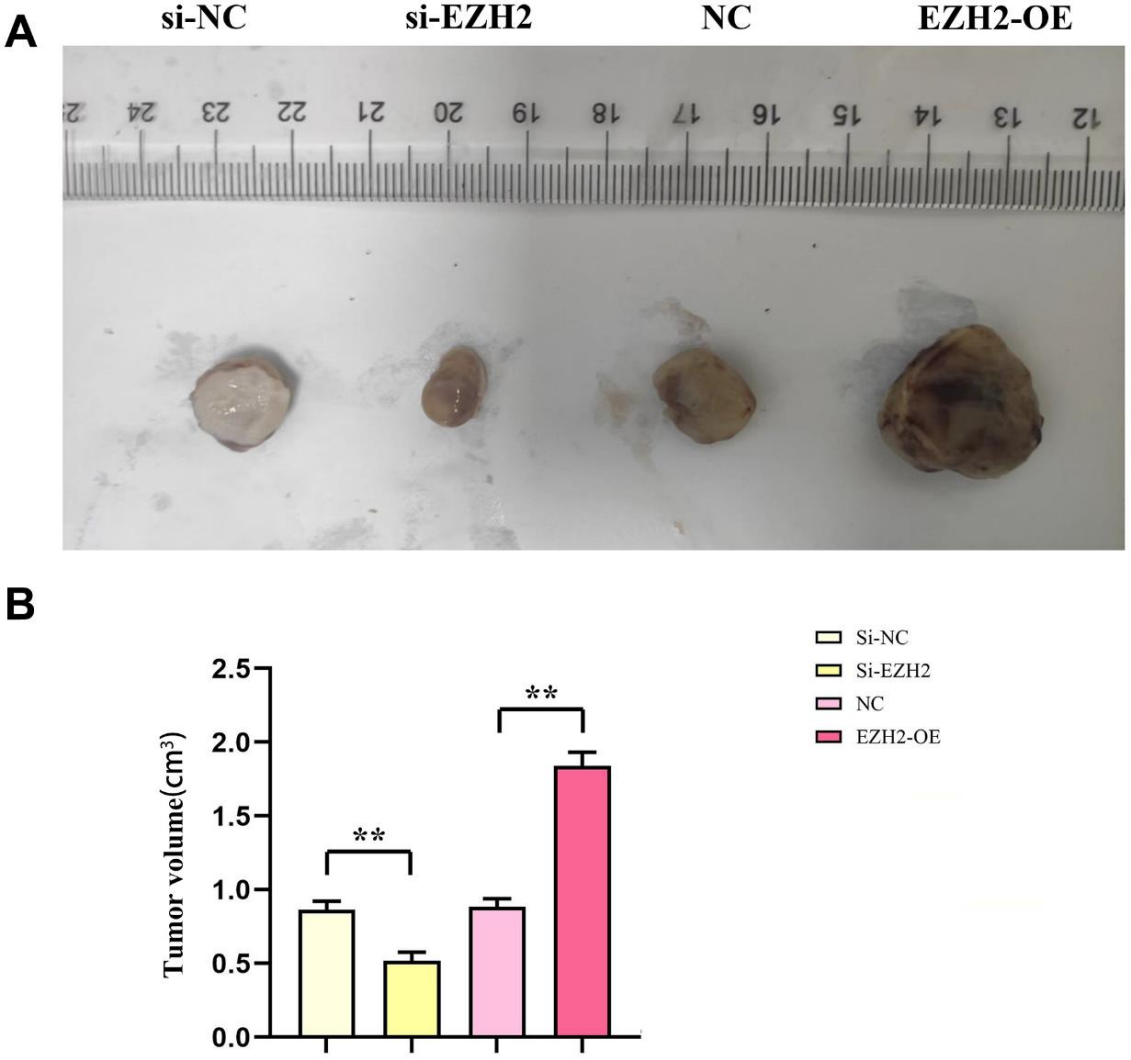
**Effect of EZH2 on the development of colorectal cancer.** (**A**) Chart of subcutaneous tumorigenesis test results in nude mice; (**B**) Statistics of subcutaneous tumorigenesis in nude mice. (**P < 0.01; N=3/Group).

The consequences of subcutaneous tumorigenesis among nude mice illustrated that cancer volume of nude mice within the si-EZH2 group was remarkably reduced than that of the si-NC group. In comparison to the NC group, the nude mouse cancer volume in the EZH2-OE group increased remarkably (*r*<0.05). It was shown in the consequences that EZH2 exerted a positive feedback impact upon colorectal cancer progression (Figure 7).

### *In vivo* Western blot results

**Figure 8:**
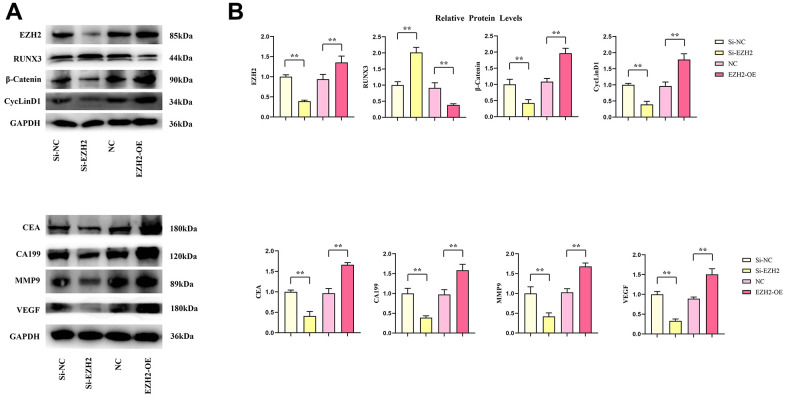
**Effects of EZH2 on the expression of RUNX3, CEA, CA199, MMP-9, VEGF, β-catenin and CyclinD1 proteins in colon cancer.** (**A**) Protein banding plots for EZH2, RUNX3, CEA, CA199, MMP-9, VEGF, β-catenin 1, CyclinD1; (**B**) Relative protein expression levels of EZH2, RUNX3, CEA, CA199, MMP-9, VEGF, β-catenin 1, CyclinD1. (** *P* < 0.01; N=3/Group).

Contrasted with the si-NC group, relative protein expressions of EZH2, β-catenin, cyclinD1, CEA, CA199, MMP9 and VEGF in the si-EZH2 group decreased significantly, whereas the relative RUNX3 protein expression was remarkably increased. Relative protein expressions of EZH2, β-catenin, cyclinD1, CEA, CA199, MMP9 and VEGF within the EZH2-OE group was remarkably increased contrasted with the NC group, whereas the relative RUNX3 protein expression was remarkably reduced (*r*<0.05) (Figure 8).

### EZH2 affects RUNX3 by regulating oxidative stress

**Figure 9:**
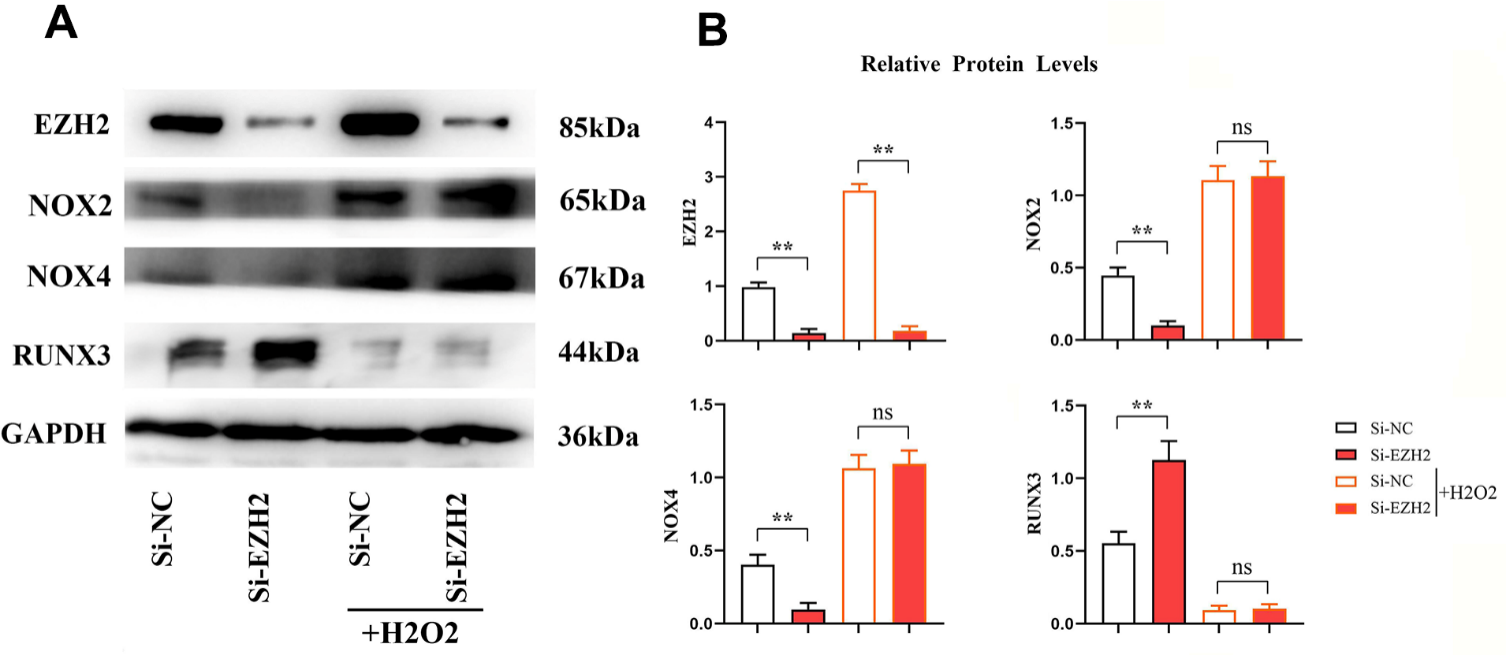
**EZH2 can regulate RUNX3 through oxidative stress.** (**A**) Protein banding plots for EZH2, RUNX3, NOX2 and NOX4; (**B**) Relative protein expression levels of EZH2, RUNX3, NOX2 and NOX4. (** *P* < 0.01; ns *P*>0.05; N=3/Group).

**Figure 10:**
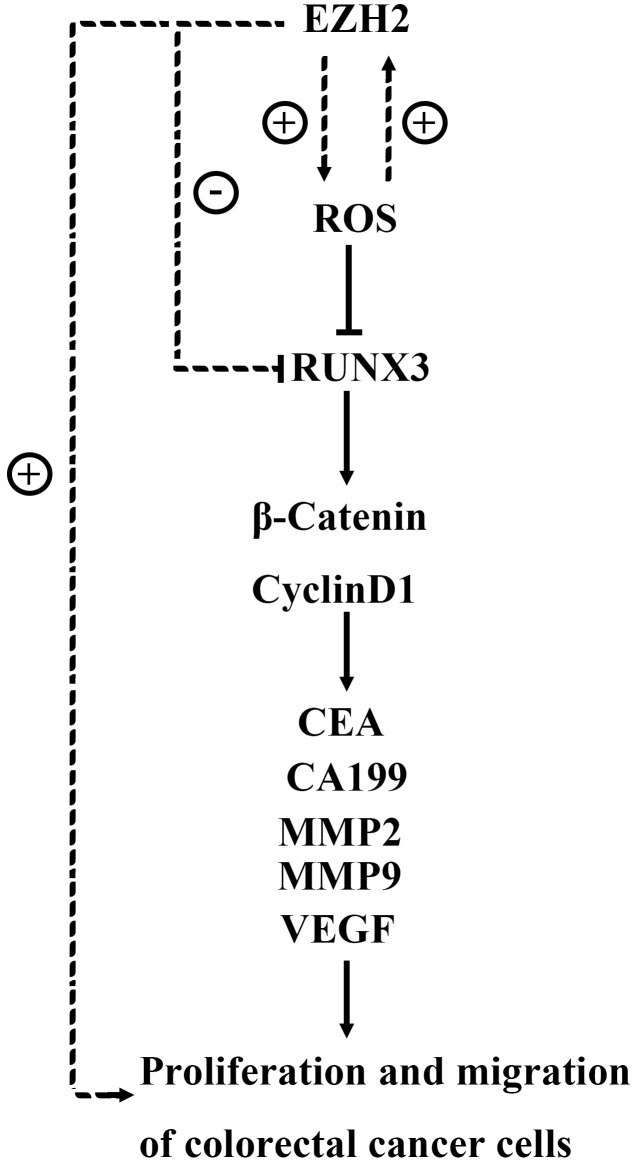
The EZH2 gene may target regulatory RUNX3 regulation via the Wnt/β-catenin signaling pathway, thereby affecting the proliferation, apoptosis, migration, and invasion of colon cancer cells.

Contrasted to the si-NC group, relative protein expressions of EZH2, NOX2, NOX4 in the si-EZH2 group reduced significantly. The RUNX3 expression was prominently increased (*r*<0.05) (Figure 9). However, after H2O2 was added, the difference in EZH2 remained constant, and the significant difference between NOX2, NOX4 and RUNX3 was eliminated. It was illustrated that EZH2 can inhibit the expression of RUNX3 through oxidative stress (Figure 10).

## DISCUSSION

Histone methyltransferase EZH2 (enhancer of zeste homolog 2) manages transcription of the anti-oncogenes and participates in transduction of cellular signaling pathways as a component of PcG protein complex 2 (PRC2) [8, 9]. Related researches have already expounded that EZH2 is implicated in cell growth, expressed highly in varieties of malignant neoplasms [10–15]. Additionally, EZH2 is closely connected to the tumor biological behaviors and patient prognosis. High expression of EZH2 is observed in lung cancer and relates to poor prognosis of lung cancer patients; both knockdown of EZH2 and application of EZH2 suppressors are capable of suppressing cell viability and cell line migrations of lung cancer and enhancing apoptosis and chemotherapy sensitivity [16]. The high EZH2 expression within invasive tissues of gastric carcinoma was linked with the invasive depth and metastasis of lymph nodes. Additionally, the EZH2 expression increased gradually with the progression of the disease [17, 18]. In pancreatic cancer, EZH2 expression is significantly elevated, and inhibition of EZH2 expression inhibits proliferation and movement of pancreatic carcinoma [19].

RUNX3 becomes a key anti-oncogene which induces apoptosis among tumor cells and plays a critical part in body progression and oncogenesis [20]. As for colon cancer, the DNA promoter area of RUNX3 is hypermethylated, and gene expression is absent. Currently, EZH2 and RUNX3 expression are discovered to be dysregulated in colorectal carcinoma tissues or cell strains. Additionally, they are involved in regulating cell proliferation, invasion, apoptosis, and various other biological processes, but the targeted regulatory relationship between them has not been clearly investigated. In this study, we found that EZH2 can mediate NOX2 and NOX4, thereby inhibiting the expression of RUNX3. As for colorectal cancer, the occurrence, progression, invasion, and metastasis are a complex procedure implicated in multiple genes. Furthermore, Wnt/β-catenin signaling pathway has already been extensively researched. Besides, its abnormal activation has also been identified in gastric carcinoma, breast carcinoma, prostate carcinoma, lung carcinoma, alongside other malignant neoplasms [21]. In the Wnt pathway, β-catenin gathers in cytoplasm and translocates into nuclei to bind to TCF/LEF, which activates target genetic transcription, such as cyclinD1. The signaling pathway is abnormally activated in neoplasm tissues. Clinically, malignant neoplasms are generally treated through suppressing the Wnt signaling pathway.

In our research, with a view to determining the specific EZH2 mechanism in colon cancer cells, we adopted RNA interference technology to reduce EZH2 expression in colon cancer cell lines and then conducted subsequent experiments. Consequences of Western blot and qRT-PCR assays revealed that EZH2 down-regulation significantly decreased EZH2 gene expression and increased RUNX3 gene expression in HCT116 and HT29 cells, indicating that EZH2 and RNX3 are negatively regulated at gene and protein levels in colon cancer, and EZH2 may interfere with colorectal cancer by up-regulating RUNX3 gene expression. The CyclinD1 and β-catenin expressions within the Wnt pathway, were significantly decreased following down-regulation of EZH2, suggesting that down-regulation of EZH2 was capable of suppressing the Wnt/β-catenin signaling pathway activation and perhaps facilitated RUNX3 expression via that pathway, thus inhibiting colorectal cancer from developing. The matrix metalloproteinase-9 (MMP-9) expression was significantly decreased after down-regulation of EZH2, showing that down-regulation of EZH2 could inhibit cancer cell movement; the vascular endothelial growth factor (VEGF) expression was prominently reduced after EZH2 down-regulation, manifesting that down-regulation of EZH2 could inhibit tumor angiogenesis, reduce nutrient supply, and suppress its development and distant metastasis. Subsequently, in the assays of cell cycles and apoptosis, we found that interference with EZH2 expression significantly increased the apoptosis percentage of colon carcinoma cells. As for colon carcinoma cells HCT116 and HT29, their proliferation, invasion and migration were prominently decreased after the EZH2 down-regulation, as shown in CCK8 and monoclonal proliferation experiments, scratch wound assay, and invasion assay. In addition, we have verified the EZH2 function on the progression of colorectal carcinoma through nude mouse tumor-bearing experiments. The above findings demonstrated that interference with EZH2 expression was capable of prominently inhibiting the malignant biological behavior of colorectal carcinoma cells.

In conclusion, in HCT116 and HT29 cell strains, knocking out EZH2 may promote expressions of proteins related to the Wnt/β-catenin signaling pathway through inhibiting RUNX3, which in turn suppresses the cancer cellular proliferation, invasion and migration while promoting apoptosis. Thus, antagonizing EZH2 has great significance in the targeted therapy of CRC. Based on the above experiments, we will continue to explore specific changes in downstream factors or pathways induced by EZH2 inhibition of RUNX3 gene expression and validate them in future animal experiments.

## MATERIALS AND METHODS

### Main reagents

McCoy’s 5A medium and calf serum were obtained from Biological Industries, Israel; CCK-8 reagents were obtained from APExBIO, USA; qRT-PCR kits were obtained from Tiangen Biotech (Beijing, China) Co., Ltd.; PCR primers were synthesized in Sangon Biotech (Shanghai, China) Co., Ltd.; Western Blot reagents were obtained from Beyotime Biotechnology, China; rabbit anti-human monoclonal antibodies (first antibodies) were obtained from Affinity Biosciences, USA; rabbit anti-human GAPDH monoclonal antibodies (first antibodies) were obtained from Hangzhou Huabio, China; horseradish peroxidase (HRP)-labeled sheep anti-rabbit polyclonal antibodies were obtained from Sera Care Life Sciences, USA; cell apoptosis assay kits were obtained from Nanjing Signalway Antibody, China; Matrigel gel were obtained from Beijing Solarbao Science and Technology Co., Ltd., China; transfection reagents, si-EZH2 and si-NC were synthesized by Ribobio, China; colon carcinoma cell strains HCT-116 were obtained from Shanghai Biomedical, China; HT29 cell strains were obtained from the Shanghai Cell Bank of the Chinese Academy of Sciences.

### Cell culture

Cell strains HCT-116 and HT29 of colon carcinoma were cultured in a 25 cm^2^ flask in McCoy’s 5A medium comprising 1% double antibodies and 10% fetal bovine serum, then brooded within one brooder (37° C, 5% CO_2_, and 95% humidity). When the density of monolayer cells reached 80-90%, cells were passaged in 0.25% trypsin solution. After transfection of si-NC and si-EZH2, H2O2 was added for treatment, respectively. During the process, all the cells were entering into the logarithmic growth stage with a good survival capability.

### Cell transfection

During the logarithmic growth stage, HCT116 and HT29 cells were inoculated within one 6-well plate. The si-RNA transfection complex was prepared following the transfection reagent instructions, and the cell suspension was added after incubated at ordinary temperature for roughly 15 min to establish the si-NC group. qRT-PCR was adopted with a view to determining the knockdown efficacy after cell transfection for 24-48 h. The si-RNA-3 with the highest knockdown efficiency (80.40 ± 7.88)% was selected from 3 stretches of si-RNAs. That was why we chose si-EZH2-3 for subsequent experiments. (Supplementary Table 1).

### qRT-PCR used to measure mRNA expressions

The overall RNAs were extracted via RNA extraction kit. 1 μl of the overall RNAs was employed with the objective to measuring the overall RNA purity and concentration on one UV spectrophotometer. Under the conditions of 50° C, 5 min → 95° C, 1 min, cDNA reverse transcription was implemented using the reverse transcription amplification kit. The reaction volume reached 20 μl (see Supplementary Table 2 concerning detailed primer sequences), alongside 7 μl Rnase Free H_2_O. The reaction process was listed: 95° C for 5 min, 1 cycle → 95° C for 10 s → 60° C for 30 s (fluorescence collection), 40 cycles. 3 replicate wells were established in the experiment. Significant consequences were defined as CT values of 15-30 and solubility curves with single peaks (peak height > 800 and peak width < 7 cells). Consequences were dissected analyzed via ΔΔCt relative quantification approach. Besides, the level of difference was expressed to be 2^-ΔΔCt^, in which ΔΔCt=experiment group (Ct target gene - Ct housekeeping gene) - control group (Ct target gene - Ct housekeeping gene).

### Western blot used to examine protein expression

Protein was obtained by digesting or lysing of total cells under ice-melting conditions. The protein concentration was determined at a wavelength of 562 nm via one BCA microplate reader. Next, the mixture was given a buffer. After five minutes of heating, the protein will be denatured. Apart from 5% concentrated gel, 10% SDS-PAGE separation gel was prepared and placed into a gel plate. Also, electrophoresis was implemented to separate the protein after solidification of the gel plate. Thereafter, the membrane was delivered to the PVDF membrane and sealed off by means of 5% skim milk powder. Afterwards, TBST was adopted with the aim of rinsing the membrane 3 times. Thereafter, as for the target protein, the first antibody was prepared in the light of the dilution proportion indicated in antibody specifications and brooded at 4° C for 12 h. Subsequently, the membrane was rinsed using TBST for 3 times (10 min every time). The secondary antibody (1:10000) was brooded at ordinary temperature for 2 h, besides, the membrane was rinsed using TBST for 3 times. Ultimately, ECL chromogenic technique was employed; quantitative dissection was implemented by means of ImageJ analysis software; for each group, the relative protein expression was expressed to be the average gray value (IOD) of the target strip. Measurements were carried out three times for all samples.

### Cell proliferation assay

During the logarithmic growth stage, HCT116 and HT29 cells were brooded in one 96-well plate (100 μl/well, about 2000 cells). Transfection was performed when the growth density reached 40-50%, and 6 replicate wells were set for every group. At 0 h, 12 h, 24 h, 36 h, and 48 h after cell transfection, 10 μl of CCK-8 solution was slowly poured into per well along the well wall, and then incubated in the cell incubator for 1 h. Then, the absorbance (OD) was measured at 450 nm with one Rayto RT-6100 ELISA plate reader. Ultimately, the curve of cell proliferation was drawn with GraphPad software based upon obtained data.

### Cell migration assay

During the logarithmic growth stage, HCT116 and HT29 cells were inoculated in one 6-well plate (1 × 10^6^ cells per well). The cells were transfected when the growth density was 40-50%, and replaced with complete culture medium after 6 h. When the cell confluence was approximately 90%, a 10 μl pipette tip was adopted with a view to making the vertical scratches on the bottom of that plate. Next, that plate was rinsed 3 times using PBS solution. Then, 2 mL of serum-free, fresh medium was placed for continuous hatching. Photographs were taken 48 h later via one Nikon TS2-S-SM inverted microscope and handled by means of ImageJ software to calculate the healing rates of cells in both groups, where the healing ratio (%) = (scratch spacing at 0 h - scratch spacing at 24/48 h) / scratch spacing at 0 h × 100%.

### Cell invasion assay

Diluted Matrigel gel (1:8) was melted overnight at 4° C. 50 μl of the gel was placed at the bottom of the upper chamber. Next, that chamber was horizontally shaken heavily and hatched at ordinary temperature for 4 h till solidified. Cells were digested and collected 24 h after transfection and inoculated into the chamber comprising the above-mentioned gel (100μl, 5 × 10^4^ cells/well). 700 μl of the total culture medium was placed into the lower chamber and hatched for 48 h. Next, that chamber was removed from the hatcher and fixed into the well comprising 700 μl of 4% paraformaldehyde for 30 min at ordinary temperature. That chamber was then removed and placed into the well at ordinary temperature to stain. Nearly 20 minutes later, that chamber was removed and rinsed using PBS. Afterwards, unmigrated cells in upper layer were cleaned using one wet cotton swab. Subsequently, that chamber was air-dried. At last, that chamber was removed, additionally, the quantity of transmembrane cells was watched and counted at random from 3 different areas of each well, and 3 repetitive wells were designated for each group.

### Cell apoptosis assay

Annexin V and PI dual staining was employed with the intention of exploring the cell apoptosis. After transfection for 24 h, it took 5 min to accumulate and centrifuge the cells at 1,000 g. Next, the supernatant was abandoned; 1 ml of pre-cooled PBS was supplemented; those cells were suspended again and centrifuged again, additionally, the supernatant was abandoned. That procedure was repeated one more time. Then, the binding buffer was diluted using DI water at 1:3, additionally, 250 μl of the solution was employed with a view to suspending the cells again. The concentration of cells was regulated to 1 × 10^6^, moreover, 100 μl of cellular suspension was pipetted into one 5 ml flow tube. Subsequently, apart from 5 ml of Annexin V/FITC, 10 ml of PI solution was supplemented. After mixed well, the tube was brooded with no light at ordinary temperature for 15 min. The final analysis was performed via flow cytometry.

### Monoclonal proliferation experiments

Cells were accumulated, counted, and assimilated via trypsin. Next, they were brooded in one 37° C brooder for rough 2 weeks till cell colonies could be seen. Then, the medium was abandoned. The cells were rinsed 3 times using PBS, immersed in carbinol for 15 min, air-dried. Additionally, they were stained for 30 min. Those cells were scanned and taken photos of so as to count the cell colonies which could be seen.

### Subcutaneous tumors in nude mice

Adult male BALB/c nude mice were from the Shanghai Laboratory Animal Research Center, fed at 25±1° C, working alternately day and night for 12 hours. Provide adequate food and water. After co-culture, each group of HCT116 cell solution was injected subcutaneously under the nude mouse` right posterior side. Additionally, cancer volume and weight were gauged and recorded once a week. After this experiment, those mice were euthanized. Additionally, tumor tissue was attained for follow-up experiments.

### Statistical analysis

Measured data was expressed to be (x ± s); t-tests of independent specimen were applied to contrast between these two groups. ImageJ 8.0 was used for image generation, and GraphPad 9.0 was for statistical graphing. As for the overall statistical comparisons, their significance was designated to *r* < 0.05.

## Supplementary Material

Supplementary Figures

Supplementary Tables

## References

[1] Sung H, Ferlay J, Siegel RL, Laversanne M, Soerjomataram I, Jemal A, Bray F. Global Cancer Statistics 2020: GLOBOCAN Estimates of Incidence and Mortality Worldwide for 36 Cancers in 185 Countries. CA Cancer J Clin. 2021; 71:209–49. 10.3322/caac.21660 33538338

[2] National Health Commission of the People’s Republic of China. [Chinese Protocol of Diagnosis and Treatment of Colorectal Cancer (2020 edition)]. Zhonghua Wai Ke Za Zhi. 2020; 58:561–85. 10.3760/cma.j.cn112139-20200518-00390 32727186

[3] Fioravanti R, Stazi G, Zwergel C, Valente S, Mai A. Six Years (2012-2018) of Researches on Catalytic EZH2 Inhibitors: The Boom of the 2-Pyridone Compounds. Chem Rec. 2018; 18:1818–32. 10.1002/tcr.201800091 30338896 PMC7410397

[4] Casciello F, Al-Ejeh F, Kelly G, Brennan DJ, Ngiow SF, Young A, Stoll T, Windloch K, Hill MM, Smyth MJ, Gannon F, Lee JS. G9a drives hypoxia-mediated gene repression for breast cancer cell survival and tumorigenesis. Proc Natl Acad Sci USA. 2017; 114:7077–82. 10.1073/pnas.1618706114 28630300 PMC5502591

[5] Lian R, Ma H, Wu Z, Zhang G, Jiao L, Miao W, Jin Q, Li R, Chen P, Shi H, Yu W. EZH2 promotes cell proliferation by regulating the expression of RUNX3 in laryngeal carcinoma. Mol Cell Biochem. 2018; 439:35–43. 10.1007/s11010-017-3133-7 28795320

[6] Balinth S, Fisher ML, Hwangbo Y, Wu C, Ballon C, Sun X, Mills AA. EZH2 regulates a SETDB1/ΔNp63α axis via RUNX3 to drive a cancer stem cell phenotype in squamous cell carcinoma. Oncogene. 2022; 41:4130–44. 10.1038/s41388-022-02417-4 35864175 PMC10132824

[7] Yuan ZL, Wu XL, Qu M, Xue J, Han L, Sun GY. [Relationship between Expression of Runt-related Transcription Factor 3 and Enhancer of zeste Homolog 2 Proteins and Sensitivity to Neoadjuvant Chemotherapy in Locally Advanced Rectal Cancer]. Zhongguo Yi Xue Ke Xue Yuan Xue Bao. 2021; 43:856–64. 10.3881/j.issn.1000-503X.13969 34980322

[8] Abdalkader L, Oka T, Takata K, Sato H, Murakami I, Otte AP, Yoshino T. Aberrant differential expression of EZH1 and EZH2 in Polycomb repressive complex 2 among B- and T/NK-cell neoplasms. Pathology. 2016; 48:467–82. 10.1016/j.pathol.2016.05.002 27311868

[9] Ferraro A, Mourtzoukou D, Kosmidou V, Avlonitis S, Kontogeorgos G, Zografos G, Pintzas A. EZH2 is regulated by ERK/AKT and targets integrin alpha2 gene to control Epithelial-Mesenchymal Transition and anoikis in colon cancer cells. Int J Biochem Cell Biol. 2013; 45:243–54. 10.1016/j.biocel.2012.10.009 23116973

[10] Lo Sardo F, Pulito C, Sacconi A, Korita E, Sudol M, Strano S, Blandino G. YAP/TAZ and EZH2 synergize to impair tumor suppressor activity of TGFBR2 in non-small cell lung cancer. Cancer Lett. 2021; 500:51–63. 10.1016/j.canlet.2020.11.037 33296708

[11] Li Z, Wang D, Lu J, Huang B, Wang Y, Dong M, Fan D, Li H, Gao Y, Hou P, Li M, Liu H, Pan ZQ, et al. Methylation of EZH2 by PRMT1 regulates its stability and promotes breast cancer metastasis. Cell Death Differ. 2020; 27:3226–42. 10.1038/s41418-020-00615-9 32895488 PMC7853151

[12] Park SH, Fong KW, Mong E, Martin MC, Schiltz GE, Yu J. Going beyond Polycomb: EZH2 functions in prostate cancer. Oncogene. 2021; 40:5788–98. 10.1038/s41388-021-01982-4 34349243 PMC8487936

[13] Patil S, Steuber B, Kopp W, Kari V, Urbach L, Wang X, Küffer S, Bohnenberger H, Spyropoulou D, Zhang Z, Versemann L, Bösherz MS, Brunner M, et al. EZH2 Regulates Pancreatic Cancer Subtype Identity and Tumor Progression via Transcriptional Repression of GATA6. Cancer Res. 2020; 80:4620–32. 10.1158/0008-5472.CAN-20-0672 32907838

[14] Ma X, Chen H, Li L, Yang F, Wu C, Tao K. CircGSK3B promotes RORA expression and suppresses gastric cancer progression through the prevention of EZH2 trans-inhibition. J Exp Clin Cancer Res. 2021; 40:330. 10.1186/s13046-021-02136-w 34666800 PMC8524915

[15] Chen Z, Du Y, Liu X, Chen H, Weng X, Guo J, Wang M, Wang X, Wang L. EZH2 inhibition suppresses bladder cancer cell growth and metastasis via the JAK2/STAT3 signaling pathway. Oncol Lett. 2019; 18:907–15. 10.3892/ol.2019.10359 31289569 PMC6539677

[16] Cao Z, Wu W, Wei H, Zhang W, Huang Y, Dong Z. Downregulation of histone-lysine N-methyltransferase EZH2 inhibits cell viability and enhances chemosensitivity in lung cancer cells. Oncol Lett. 2021; 21:26. 10.3892/ol.2020.12287 33240432 PMC7681225

[17] Sun B, Lin Y, Wang X, Lan F, Yu Y, Huang Q. Single Nucleotide Polymorphism of the Enhancer of Zeste Homolog 2 Gene rs2072408 is Associated with Lymph Node Metastasis and Depth of Primary Tumor Invasion in Gastric Cancer. Clin Lab. 2016; 62:2099–105. 10.7754/Clin.Lab.2016.160302 28164661

[18] Gan L, Xu M, Hua R, Tan C, Zhang J, Gong Y, Wu Z, Weng W, Sheng W, Guo W. The polycomb group protein EZH2 induces epithelial-mesenchymal transition and pluripotent phenotype of gastric cancer cells by binding to PTEN promoter. J Hematol Oncol. 2018; 11:9. 10.1186/s13045-017-0547-3 29335012 PMC5769437

[19] Zhou X, Gao W, Hua H, Ji Z. LncRNA-BLACAT1 Facilitates Proliferation, Migration and Aerobic Glycolysis of Pancreatic Cancer Cells by Repressing CDKN1C via EZH2-Induced H3K27me3. Front Oncol. 2020; 10:539805. 10.3389/fonc.2020.539805 33072570 PMC7538708

[20] Silva KA, Dong J, Dong Y, Dong Y, Schor N, Tweardy DJ, Zhang L, Mitch WE. Inhibition of Stat3 activation suppresses caspase-3 and the ubiquitin-proteasome system, leading to preservation of muscle mass in cancer cachexia. J Biol Chem. 2015; 290:11177–87. 10.1074/jbc.M115.641514 25787076 PMC4409274

[21] Tan BL, Norhaizan ME, Huynh K, Heshu SR, Yeap SK, Hazilawati H, Roselina K. Water extract of brewers’ rice induces apoptosis in human colorectal cancer cells via activation of caspase-3 and caspase-8 and downregulates the Wnt/β-catenin downstream signaling pathway in brewers’ rice-treated rats with azoxymethane-induced colon carcinogenesis. BMC Complement Altern Med. 2015; 15:205. 10.1186/s12906-015-0730-4 26122204 PMC4487214

