# Supplementary material for: Down-regulation of EZH2 genes targeting RUNX3 affects proliferation, invasion, and metastasis of human colon cancer cells by Wnt/β-catenin signaling pathway
Source: Aging (Albany NY). 2023 Dec 2;15(23):13655–68. doi: 10.18632/aging.205197 (PMC10756104; doi:10.18632/aging.205197)
Supplement: Supplementary Figures [file aging-15-205197-s001.pdf]

SUPPLEMENTARY FIGURES

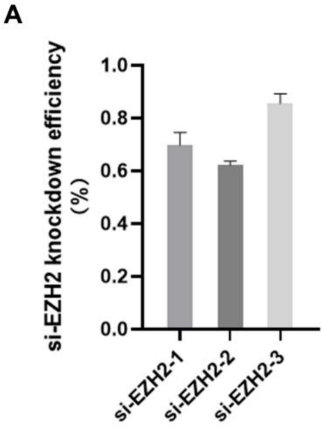

Supplementary Figure 1. si-EZH2 knockdown efficiency (%) (*P*<0.05). (**A**) Knockout efficiency of si-EZH2-1, si-EZH2-2 and si-EZH2-3 on EZH2.

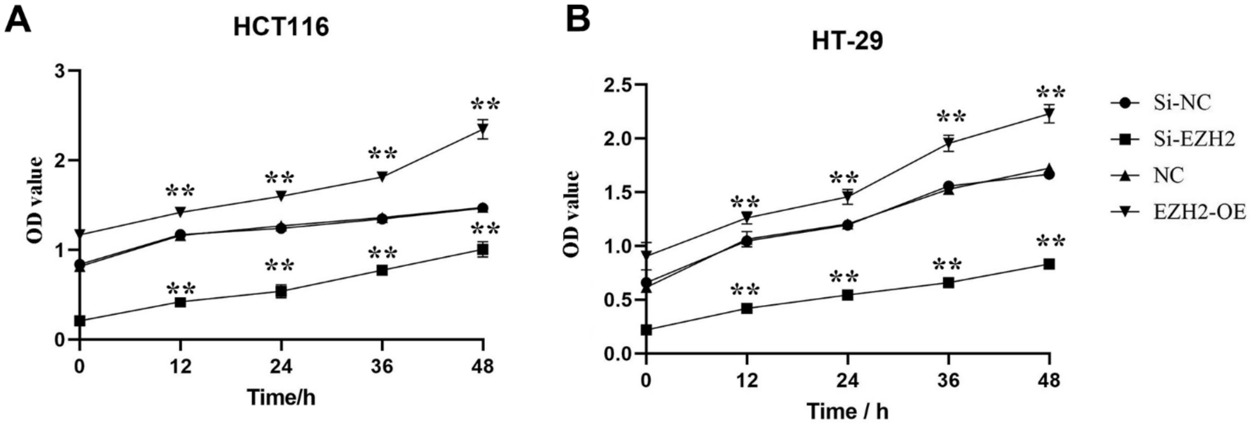

Supplementary Figure 2. Cell proliferation curve (OD value). (**A**) CCK8 shows the effect of EZH2 on the proliferative capacity of HCT116 cells; (**B**) CCK8 shows the effect of EZH2 on the proliferative capacity of HT29 cells. (****P* < 0.001, ***P* < 0.01).
