# Supplementary material for: Down-regulation of EZH2 genes targeting RUNX3 affects proliferation, invasion, and metastasis of human colon cancer cells by Wnt/β-catenin signaling pathway
Source: Aging (Albany NY). 2023 Dec 2;15(23):13655–68. doi: 10.18632/aging.205197 (PMC10756104; doi:10.18632/aging.205197)
Supplement: Supplementary Tables [file aging-15-205197-s002.pdf]

SUPPLEMENTARY TABLES

Supplementary Table 1. The sequences of si-EZH2.

siRNA	Sequences
EZH2-1	5'-GCUGGAAUCAAAGGAUACA-3'
EZH2-2	5'-CCAUGUUACAACUAUCAA-3'
EZH2-3	5'-GACU CUGAAUGCAGUUGCU-3'

Supplementary Table 2. The sequences of PCR primers.

GENES	Primer (from 5' to 3')
EZH2	F:5'-TTGTTGGCGGAAGCGTGTAAAATC-3'<br>R:5'-TCCCTAGTCCCGCGCAATGAGC-3'
RUNX3	F:5'-AGGCAATGACGAGAACTACTCC-3'<br>R:5'-CGAAGGTCGTTGAACCTGG-3'
β-catenin	F:5'-GCCCTAGCTGCCAACAGTAGT-3'<br>R:5'-GAAGATGAACGCTGTTTCTCG-3'
CyclinD1	F:5'-GCTGCGAAGTGGAAACCATC-3'<br>R:5'-CCTCCTTCTGCACACATTTGAA-3'
CEA	F:5'-TAAGTGTTGACCACAGCGACCC-3'<br>R:5'-GTTCCCATCAATCAGCCAAGAA-3'
CA199	F:5'-TCTAACCCATCCCCGCAGTA-3'<br>R:5'-AGCTGTTGCAAATGCAGTCTT-3'
MMP9	F:5'-AAGGGTACAGCCTGTTCCTGGT-3'<br>R:5'-CTGGATGCCGTCTATGTCGTCT-3'
VEGF	F:5'-TGGCAGCGAGAAACATTCTTTTAT-3'<br>R:5'-CAGCAATACTCCGTAAGACCACAC-3'
β-actin	F:5'-TACATGGCTGGGGTGTTGAA-3'<br>R:5'-AAGAGAGGCATCCTCACCCT-3'
